# Supplementary material for: Evaluation of the Bioactive Compounds of Apis mellifera Honey Obtained from the Açai (Euterpe oleracea) Floral Nectar
Source: Molecules. 2024 Sep 25;29(19):4567. doi: 10.3390/molecules29194567 (PMC11477771; doi:10.3390/molecules29194567)
Supplement: Supplementary file 1 [file molecules-29-04567-s001.zip › molecules-3146245-supplementary.pdf]

Figure S1. GC-MS Chromatogram of Açaí Honey AH2, from Breu Branco (Pará, Brazil).

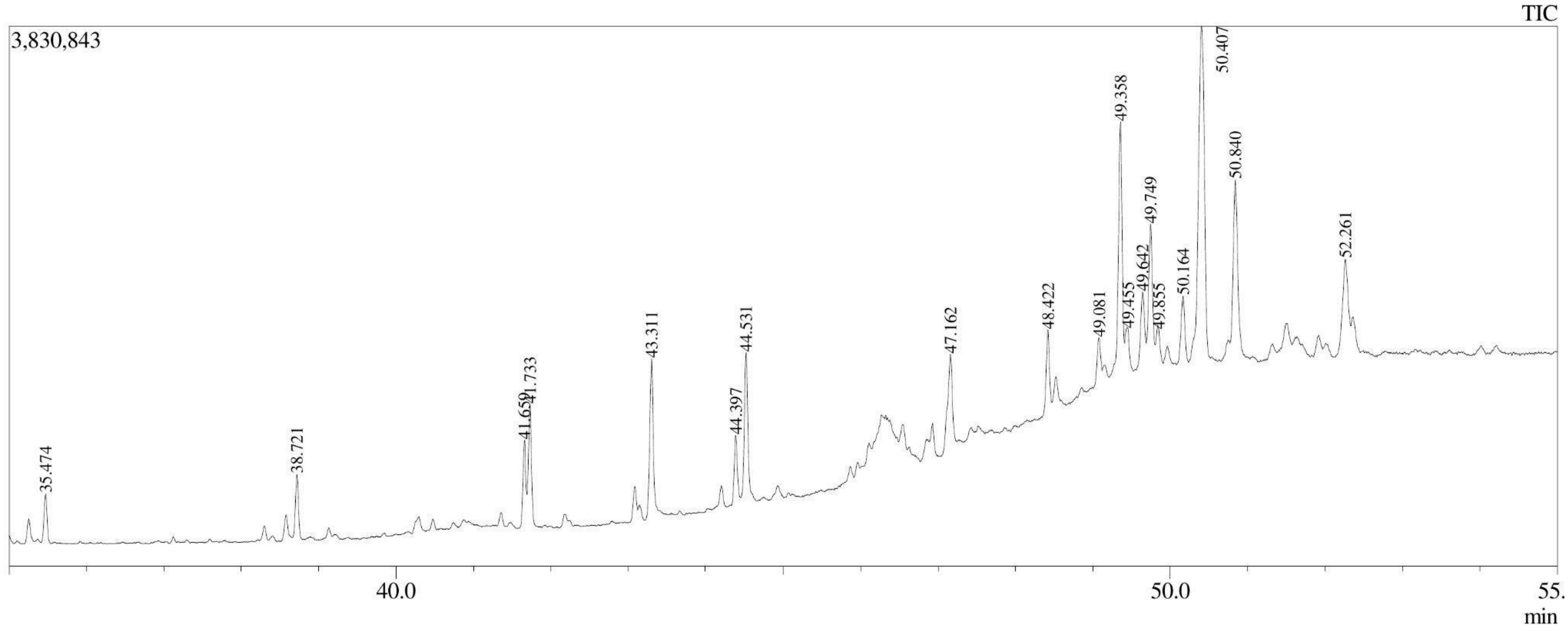

Table S1. Identified Compounds by GC-MS in Açaí Honey AH2, from Breu Branco (Pará, Brazil).

Peak#	R.Time	MI	Fragments (%)	Mark	Name
1	31.92	296	55 (100); 69(13.6); 264 (75)		Methyl 9-octadecenoate
2	35.47	282	57 (100); 71 (82.6); 85 (54.8)		Eicosane
3	38.72	282	57 (100); 71 (82.6); 85 (54.8)		Eicosane
4	41.66	354	83 (100); 97 (94.5); 69 (80.4)		n-tetracosanol-1
5	41.73	354	83 (100); 97 (94.5); 69 (80.4)		n-tetracosanol-1
6	43.31	380	57 (100); 71 (82.6); 85 (54.8)		2-methyl-n-hexacosane
7	44.40	396	57 (100); 97 (88.0); 83 (85,0)		heptacosanol
8	44.53	380	57 (100); 71 (82.3); 43 (60.8)	V	2-Methylhexacosane
9	47.16	450	57(100); 71 (83.1); 85 (71.7)		Dotriacontane
10	48.42	412	55 (100); 314 (95.8); 81 (95.3)		stigmasta-5,24(28)-dien-3-ol
11	49.08	324	82 (100); 55(82.6); 96 (54.8)		13-Docosen-1-ol
12	49.36	396	55 (100); 97 (82.6); 83 (54.8)		1-Heptacosanol
13	49.45	396	55 (100); 97 (82.6); 83(54.8)	V	1-Heptacosanol
14	49.64	414	57 (100); 43 (82.6); 71 (54.8)		stigmast-5-en-3.β.-ol
15	49.75	468	218 (100); 203 (82.6); 189 (54.8)	V	β-Amyrin acetate
16	49.85	412	314(100); 55 (82.6); 281 (54.8)	V	stigmasta-5,24(28)-dien-3-ol
17	50.16	412	218 (100); 203 (82.6); 189 (54.8)		α-amyrine
18	50.41	468	218 (100); 95 (82.6); 109 (54.8)		lupeol acetate
19	50.84	-	218 (100); 95 (82.6); 135 (54.8)		no identified
20	52.26	-	218 (100); 43 (82.6); 189 (54.8)		no identified

Figure S2. GC-MS Chromatogram of Açaí Honey AH3, from Santa Maria (Pará, Brazil).

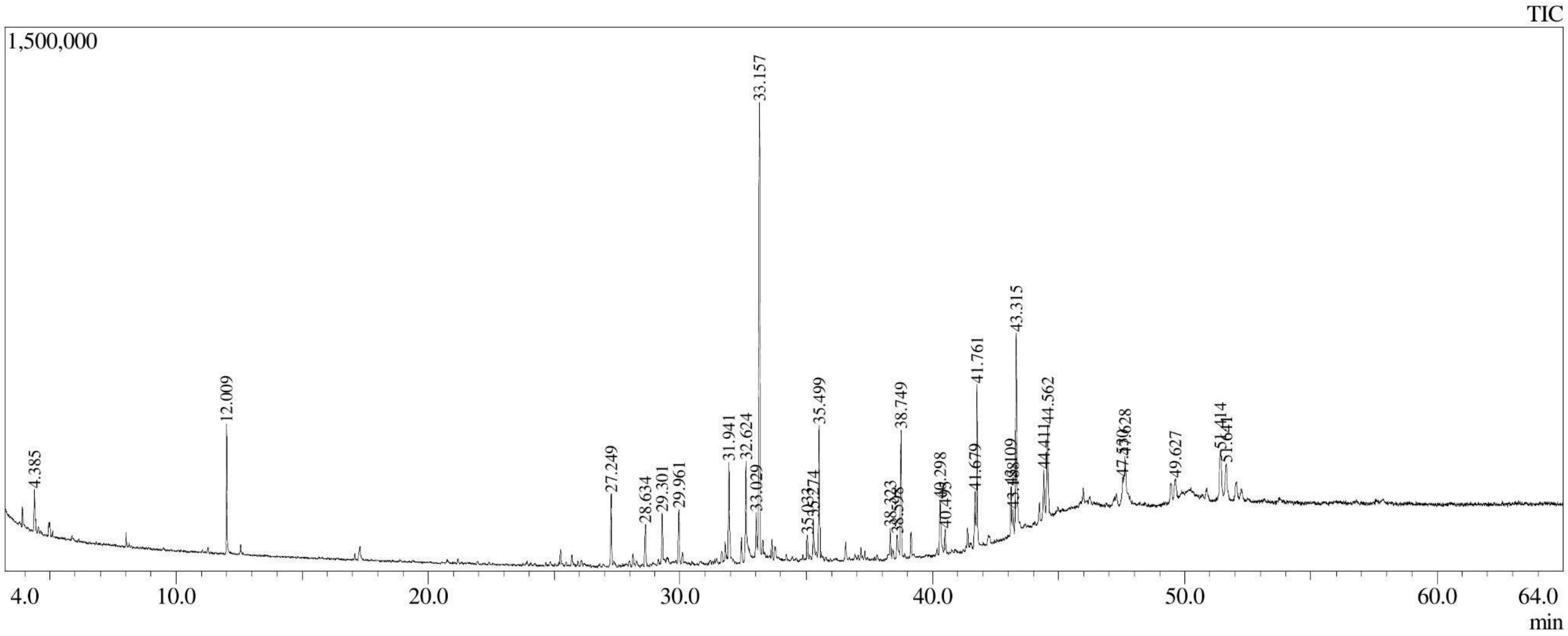

Table S2. GC-MS Chromatogram of Açaí Honey AH3, from Santa Maria (Pará, Brazil).

Peak#	R.Time	MI	Fragments (%)	Mark	Name
1	4.38	140	84 (100); 57(82.5); 69 (75.8)		1,2,3,4,5-pentamethylcyclopentane
2	12.01	126	97 (100); 41 (69.5); 126(51.3)		5-hydroxymethylfurfural
3	27.25	278	149 (100); 57 (23.2); 41(10.4)		1,2-benzenedicarboxylicacid, bis(2-methylpropyl) ester
4	28.63	410	74 (100); 87(69.7); 277(43.5)		methylhexacosanoate
5	29.30	256	73 (100); 43 (80.1); 60(72.9)		hexadecanoic acid
6	29.96	284	88 (100); 101 (61.0); 43 (35.4)		ethylhexacosanoate
7	31.94	296	55 (100); 69(72.0); 74(65.1)	V	Methyl 9-octadecenoate
8	32.62	282	55 (100); 69 (85.6); 41(64.2)		Oleic Acid
9	33.03	266	67(100); 81(88.3); 95(60.4)		9,12-Octadecadien-1-ol
10	33.16	310	55(100); 69 (79.0); 83 (62.3)	V	Ethyl Oleate
11	35.03	326	97 (100); 83(96.7); 69 (96.3)		Behenic alcohol
12	35.27	326	83 (100); 57 (94.8); 69 (90.9)		Behenic alcohol
13	35.50	296	57 (100); 71(82.1); 43(59.9)		Heneicosane
14	38.32	266	97 (100); 57 (99.5); 83 (94.1)		1-Nonadecene
15	38.60	326	83(100); 97(98.3); 57 (97.3)		Behenic alcohol
16	38.75	296	57(100); 71 (84.3); 43 (58.5)	V	Heneicosane
17	40.30	380	57(100); 71 (77.1); 43 (57.6)		2-Methylhexacosane
18	40.50	296	57 (100); 43 (69.9); 71 (62.6)	V	Octadecane, 1-(ethenyloxy)
19	41.68	326	97 (100); 83 (96.9); 69 (87.3)		Behenic alcohol
20	41.76	296	57 (100); 71 (86.7); 85 (57.8)	V	Heneicosane
21	43.11	426	185 (100); 57(60.2); 71 (57.8)		Decanedioic acid, bis(2-ethylhexyl) ester
22	43.19	408	57 (100); 71(89.3); 85 (57.8)		2-methyloctacosane
23	43.31	344	57 (100); 71 (70.3); 85 (57.8)		Batilol
24	44.41	490	57 (100); 43 (82.5); 97 (57.8)		17-Pentatriacontene
25	44.56	408	57 (100); 71 (87.2); 43 (57.8)		2-Methylhexacosane

Table S2. GC-MS Chromatogram of Açaí Honey AH3, from Santa Maria (Pará, Brazil).

continuous

Peak#	R.Time	MI	Fragments (%)	Mark	Name
26	47.53	410	97 (100); 83 (92.7); 69 (57.8)		Octacosanol
27	47.62	282	57 (100); 71 (86.7); 85 (57.8)		Eicosane
28	49.62	536	57 (100); 107 (99.3); 71 (57.8)		1-Heptatriacotanol
29	51.41	410	57 (100); 97 (99.8); 83 (57.8)		Octacosanol
30	51.64	414	43 (100); 107 (98.9); 145 (57.8)		Sitosterol

Figure S3. GC-MS Chromatogram of Timbó honey (Rio Grande do Sul, Brazil).

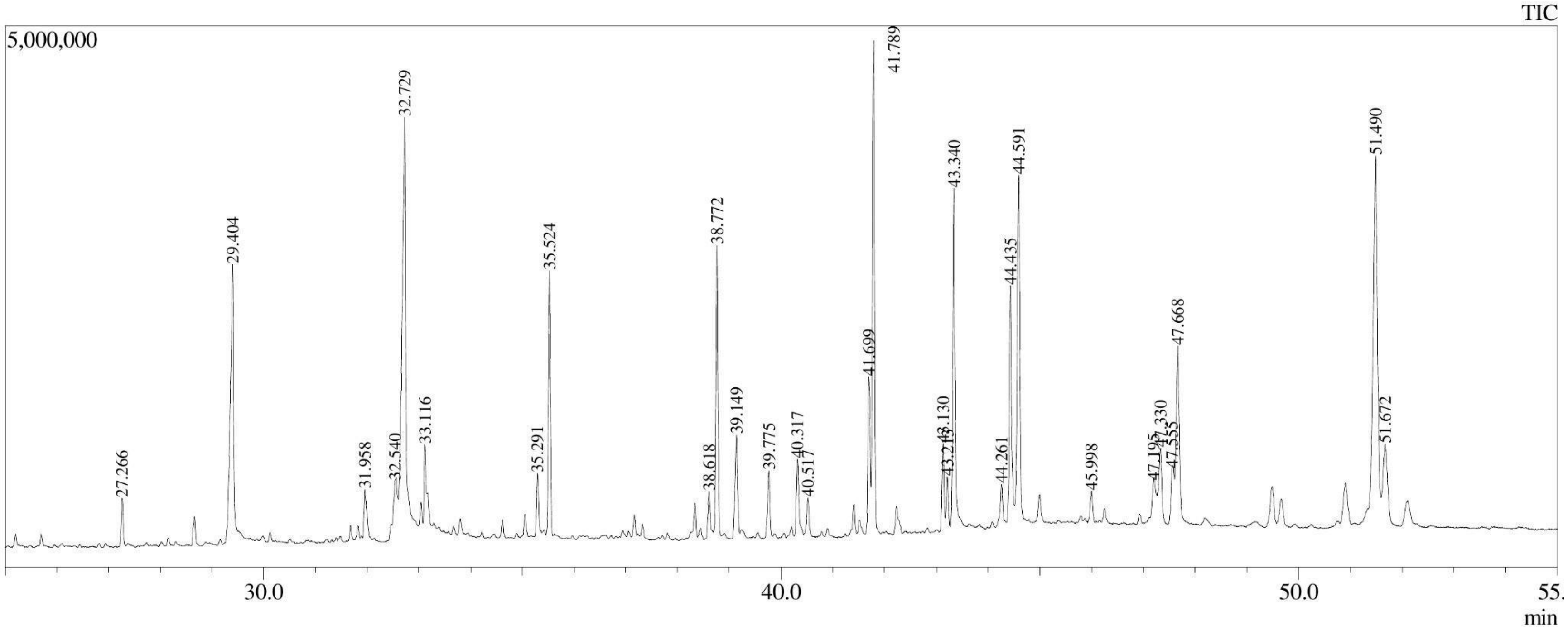

Table S3. GC-MS Chromatogram of Timbó honey (Rio Grande do Sul, Brazil).

Peak#	R.Time	MI	Fragments (%)	Mark	Name
1	3.96	90	42 (100); 43 (89.0); 72 (75)		Glyceraldehyde
2	27.26	278	149(100); 57(23.2); 41 (54.8)		1,2-Benzenedicarboxylic acid
3	29.40	256	73 (100); 43 (83.0); 60 (54.8)		n-Hexadecanoic acid
4	31.95	296	55(100); 69 (71.2); 74 (80.4)		9-Octadecenoic acid, methyl ester,
5	32.54	280	67 (100); 81 (92.6); 95 (80.4)		9,12-Octadecadienoic acid
6	32.72	282	55(100); 69 (80.5); 83 (54.8)	V	Oleic Acid
7	33.11	284	73 (100); 43 (89.8); 57 (83.9)		Octadecanoic acid
8	35.29	326	83 (100); 69 (87.0); 97 (87.0)		Behenic alcohol
9	35.52	296	57 (100); 71(81.6); 85 (55.8)		Heneicosane
10	38.61	354	83 (100); 57 (97.3); 97 (87.3)		n-Tetracosanol-1
11	38.77	296	57 (100); 71 (81.2); 85 (57.3)	V	Heneicosane
12	39.14	224	149 (100); 171 (40.8); 131 (40.4)		9-t-Butyltricyclo[4.2.1.1(2,5)]decane-9,10-diol
13	39.77	-	130 (100); 171(87.3); 41(74.8)		No identified
14	40.31	380	57 (100); 71 (78.3); 43 (54.7)		2-Methylhexacosane
15	40.51	242	57 (100); 43 (58.1); 83 (55.6)		Oxirane, [(dodecyloxy)methyl]
16	41.69	396	83 (100); 97 (94.4); 57(90.8)		1-Heptacosanol
17	41.78	352	57 (100); 71 (83.3); 85 (59.5)	V	Pentacosane
18	43.13	426	185 (100); 57 (52.2); 71 (47.2)		Decanedioic acid, bis(2-ethylhexyl) ester
19	43.21	562	57 (100); 71 (84.4); 85 (62.8)	V	Tetracontane
20	43.34	344	57 (100); 71 (54.2); 85 (45.9)		Batilol
21	44.26	396	57 (100); 97 (99.0); 83 (98.9)		Heptacosanol
22	44.43	490	57 (100); 97 (99.7); 83 (99.1)		17-Pentatriacontene
23	44.59	618	57 (100); 71 (80.7); 85 (60,5)	V	Tetratetracontane
24	45.99	562	57 (100); 71 (80.7); 85 (60,5)		Tetracontane
25	47.19	396	57 (100); 97 (99.0); 83 (98.9)		Heptacosanol

Table S3. GC-MS Chromatogram of Timbó honey (Rio Grande do Sul, Brazil).

continuous

**Peak#**	**R.Time**	**MI**	**Fragments (%)**	**Mark**	**Name**
26	47.33	382	57 (100); 97 (99.0); 83 (98.9)	V	Hexacosanol
27	47.55	594	97 (100); 83 (98.0); 69 (70.8)		Tetracontane-1,40-diol
28	47.66	562	57 (100); 71 (80.7); 85 (60,5)	V	Tetracontane
29	51.49	382	57 (100); 97 (99.0); 83 (98.9)		1-Hexacosanol
30	51.67	414	43 (100); 107 (98.9); 145 (57.8)	V	Sitosterol

Figure S4. GC-MS Chromatogram of Mangue honey (Pará, Brazil).

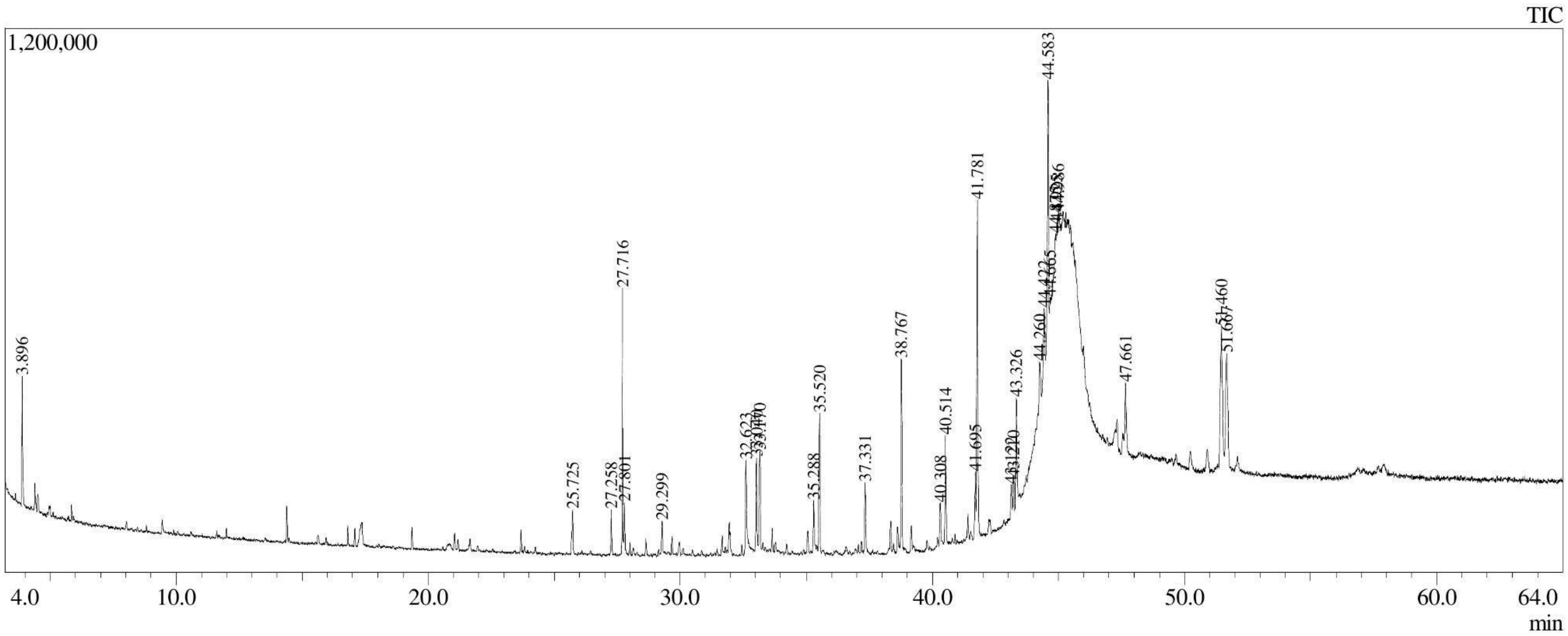

Table S4. GC-MS Chromatogram of Mangue honey (Pará, Brazil).

Peak#	R.Time	MI	Fragments (%)	Mark	Name
1	3.89	90	42 (100); 43 (89.0); 72 (75)		Glyceraldehyde
2	25.75	278	149(100); 57(23.2); 41 (54.8)		1,2-Benzenedicarboxylic acid
3	27.25	278	149 (100); 57 (71.2); 223 (80.4)		1,2-Benzenedicarboxylic acid, bis(2 methyl
4	27.71	152	79 (100); 71 (92.6); 41 (98.4)		Spiro[bicyclo[3.1.1]heptane-2,2'-oxirane],
5	27.80	222	85 (100); 95 (92.6); 41 (80.4)	V	Decahydro-1,5,5,8a-tetramethyl-1,4 methanoazulen-9-ol
6	29.29	256	73 (100); 43 (85.9); 57 (76.4)		n-Hexadecanoic acid
7	32.62	282	55 (100); 69 (82.3); 41(63.9)		Oleic Acid
8	33.04	308	67 (100); 81 (92.6); 95 (80.4)		9,12-Octadecadienoic acid, ethyl ester
9	33.17	310	55(100); 69 (79.0); 83 (62.3)	V	Ethyl Oleate
10	35.28	326	83 (100); 69 (93.3); 57 (90.9)		Behenic alcohol
11	35.52	296	57 (100); 71 (84.7); 43 (57.0)		Heneicosane
12	37.33	298	57 (100); 43 (62.3); 71(59.3)		Oxirane, [(dodecyloxy)methyl]
13	38.76	296	57 (100); 71 (84.7); 43 (57.0)	V	Heneicosane
14	40.30	380	57 (100); 71 (82.3); 43 (60.8)		2-Methylhexacosane
15	40.51	298	57 (100); 43 (62.3); 71(59.3)		Oxirane, [(dodecyloxy)methyl]-
16	41.69	326	83 (100); 69 (93.3); 57 (90.9)		Behenic alcohol
17	41.78	352	57 (100); 71 (83.3); 85 (59.5)	V	Pentacosane
18	43.12	426	185 (100); 57 (52.2); 112 (47.2)		Decanedioic acid, bis(2-ethylhexyl) ester
19	43.21	548	57 (100); 71 (89.2); 85 (60.2)	V	1-Iodotriacontane
20	43.32	298	57 (100); 43 (62.3); 71(59.3)		Oxirane, [(dodecyloxy)methyl]
21	44.26	396	97 (100); 57 (99.3); 83(91.4)	V	1-Heptacosanol
22	44.42	490	57 (100); 43 (82.5); 97 (57.8)		17-Pentatriacontene
23	44.58	618	57 (100); 71 (83.3); 43 (56.8)		Tetratetracontane
24	44.66	340	45 (100); 60 (80.7); 43 (60,5)	V	Propyleneglycol monoleate
25	44.87	282	55 (100); 69 (99.0); 83 (98.9)	V	9-Octadecenoic acid
26	44.92	298	155 (100); 55 (89.0); 69 (87.4)	V	Oxiraneoctanoic acid, 3-octyl-, methyl ester

Table S4. GC-MS Chromatogram of Mangue honey (Pará, Brazil).

continuous

**Peak#**	**R.Time**	**MI**	**Fragments (%)**	**Mark**	**Name**
27	44.98	380	57 (100); 71 (82.3); 43 (60.8)	V	2-Methylhexacosane
28	47.66	548	57 (100); 71 (89.2); 85 (60.2)	V	1-Iodotriacontane
29	51.46	382	82 (100); 96 (97.2); 85 (81.0)		1-Hexacosanol
30	51.66	414	43 (100); 107 (98.9); 145 (57.8)	V	Sitosterol

Figure S5. GC-MS Chromatogram of Cipó-Uva honey, from Distrito Federal (Brasília, Brazil).

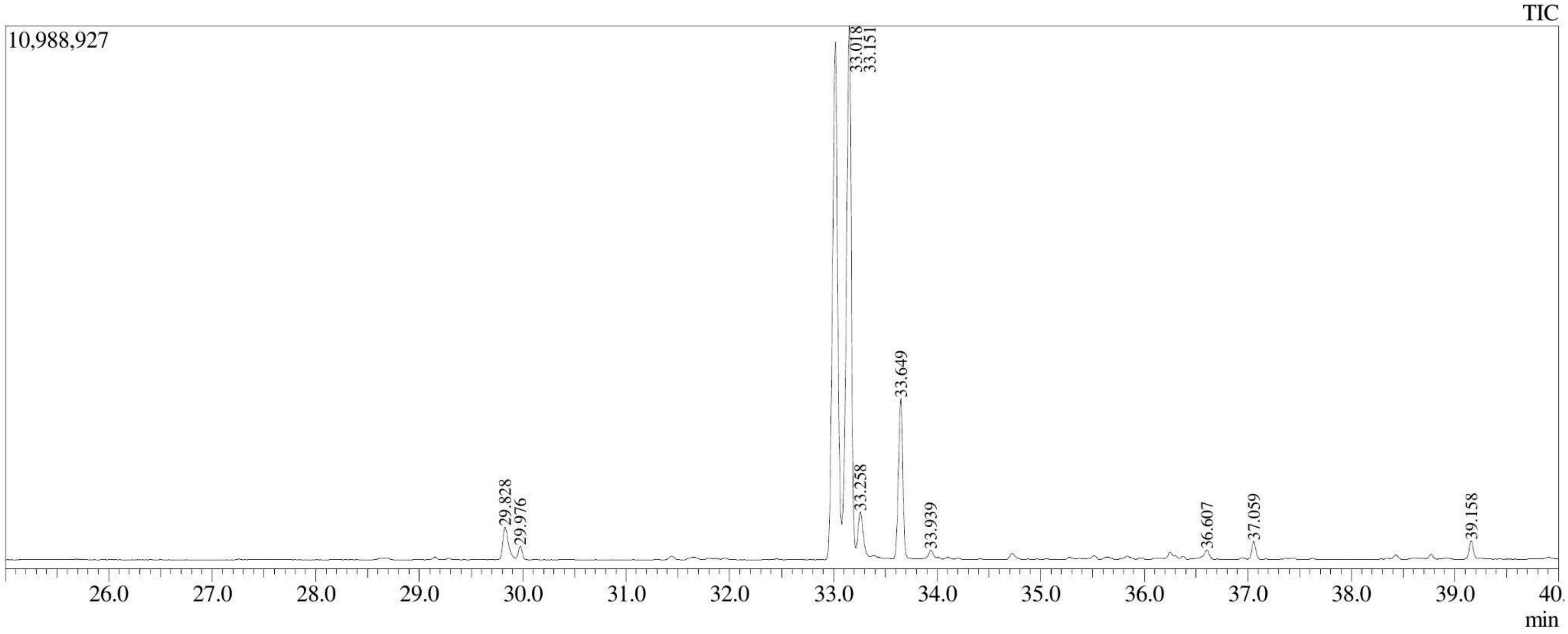

Table S5. GC-MS Chromatogram of Cipó-Uva honey, from Distrito Federal (Brasília, Brazil).

Peak#	R.Time	MI	Fragments (%)	Mark	Name
1	19.35	200	55 (100); 88 (90.4); 83 (72.1)		Ethyl 9-oxononanoate
2	29.82	284	88 (100); 101 (58.7); 43 (29.4)		Hexadecanoic acid, ethyl ester
3	29.97	284	88 (100); 101 (59.1); 43 (29.1)	V	Hexadecanoic acid, ethyl ester
4	33.01	308	67 (100); 81 (93.1); 95 (65,0)		Linoleic acid ethyl ester
5	33.15	310	55 (100); 69 (81.4); 83 (65.8)	V	Ethyl 9-octadecenoate
**6**	33.25	310	55 (100); 69 (82.0); 83 (58.6)	V	Ethyl 9-octadecenoate
7	33.64	312	88 (100); 101 (63.0); 43 (31.7)		Octadecanoic acid, ethyl ester
8	33.93	666	73 (100); 355 (51.7); 147 (49.9)		Cyclononasiloxane, octadecamethyl-
9	36.60	310	55 (100); 69 (73.8); 83 (64.8)		9-Octadecenoic acid, ethyl ester (ethyl 9-octadecenoate
10	37.05	340	88 (100); 101(65.4); 43 (35.1)		Eicosanoic acid, ethyl ester
11	39.15	390	149 (100); 57 (30.0); 167 (29.2)		Bis(2-ethylhexyl) phthalate
12	40.88	361	249 (100); 232 (82.9); 70 (83.5)		Octocrylene
13	41.77	562	57 (100); 71 (84.0) 85 (57.7)		Tetracontane
14	43.32	410	69 (100); 81 (59.8); 41 (23.0)	V	Squalene
15	44.58	506	57 (100); 71 (83.6); 85 (61.7)		Hexatriacontane

Figure S6. GC-MS Chromatogram of Aroeira honey, from Belo Horizonte (Minas Gerais, Brazil).

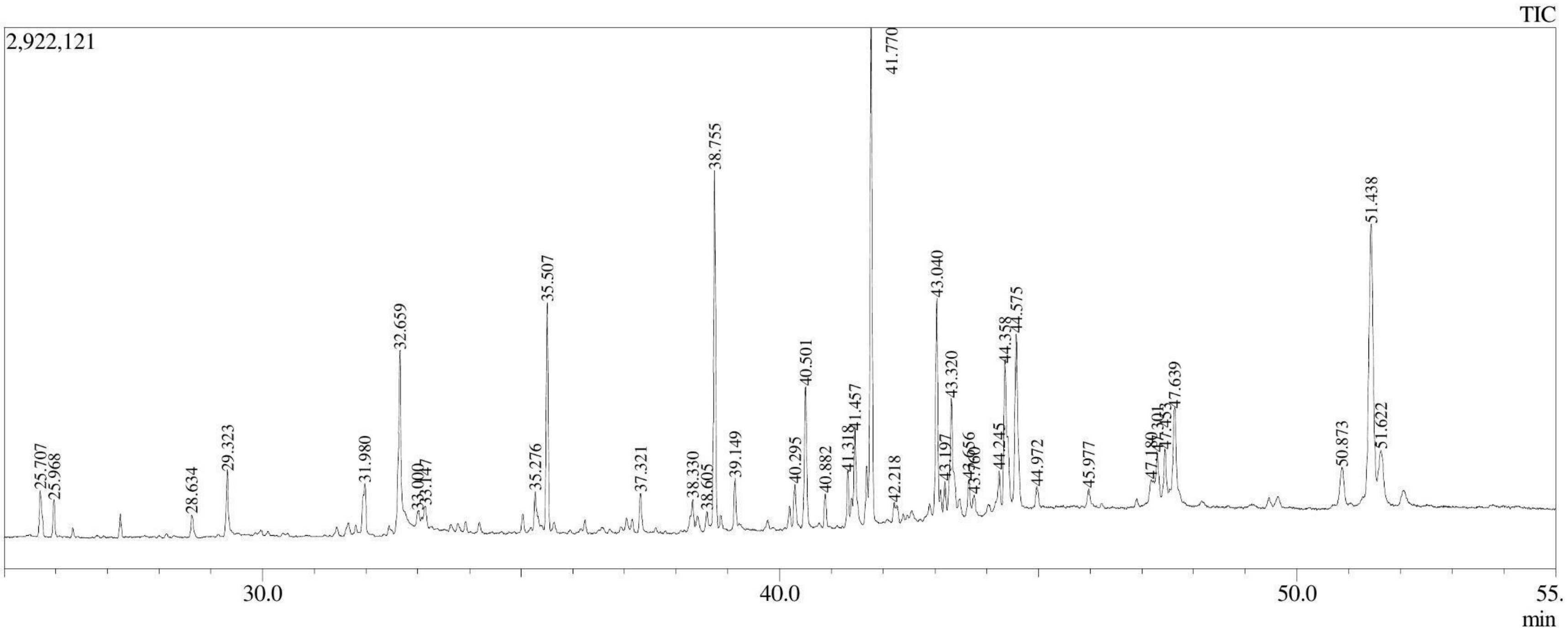

Table S6. GC-MS Chromatogram of Aroeira honey, from Belo Horizonte (Minas Gerais, Brazil).

Peak#	R.Time	MI	Fragments (%)	Mark	Name
1	17.34	152	135 (100); 152 (75.9); 77 (26.4)	MI	4-Methoxybenzoic acid
2	19.19	166	121 (100); 166 (23.9); 77 (15.9)		4-Methoxyphenyl) acetic acid
3	25.70	222	124 (100); 43 (43.5); 166 (10.3)		2-Cyclohexen-1-one
4	25.96	145	144 (100); 116 (41.4); 89 (33.9)		Indol-3-carboxaldehyde
5	28.63	410	74 (100); 87 (66.6); 43 (29.9)		Methyl hexacosanoate
6	29.32	256	73 (100); 43 (85.9); 57 (76.4)		n-Hexadecanoic acid
7	31.98	282	57 (100); 71 (78.0); 43 (62.6)		Eicosane
8	32.65	282	55 (100); 69 (82.3); 41(63.9)		Oleic Acid
9	33.00	266	67 (100); 81 (90.8); 95 (58.7)	V	1,8,11-Heptadecatriene
10	33.14	296	55 (100); 69 (84.1); 88 (66.2)	V	Ethyl heptadec-9-enoate
11	35.27	326	83 (100); 69 (93.3); 57 (90.9)		Behenic alcohol
12	35.50	282	57 (100); 71 (83.2); 43 (59.1)	V	Eicosane
13	37.32	298	57 (100); 43 (62.3); 71(59.3)		Oxirane, [(dodecyloxy)methyl]
14	38.330	322	97 (100); 83 (96.3); 57 (96.0)		9-Tricosene
15	38.605	326	83 (100); 57 (99.8); 97 (91.6)		Behenic alcohol
16	38.755	296	57 (100); 71 (84.7); 43 (57.0)	V	Heneicosane
17	39.149	390	148 (100); 167 (29.4); 57 (27.7)		Bis(2-ethylhexyl) phthalate
18	40.295	618	57 (100); 71 (83.3); 43 (56.8)	V	Tetratetracontane
19	40.501	298	57 (100); 43 (63.1); 71 (60.2)		Oxirane, [(dodecyloxy)methyl]
20	40.882	361	249(100); 232 (85.4); 204 (78,7)		Octocrylene
21	41.318	279	279 (100); 264 (49); 204 (12.0)		2-(4-Methoxyphenyl)-8H-thieno[2,3-b]indole
22	41.770	618	57 (100); 71 (89.2); 85 (60.2)		Tetratetracontane
23	42.218	548	71 (100); 57 (90.1); 85 (61.5)		Triacontane, 1-iodo
24	43.040	-	135 (100); 296 (90.1); 239 (61.5)		No identified
25	43.19	548	57 (100); 71 (89.2); 85 (60.2)	V	1-Iodotriacontane
26	43.32	298	57 (100); 43 (62.3); 71(59.3)	V	Oxirane, [(hexadecyloxy)methyl]
27	43.65	418	149 (100); 309 (41.3); 71(31.3)		Phthalic acid, bis(7-methyloctyl) ester
28	43.76	418	149 (100); 309 (41.3); 71(31.3)	V	Phthalic acid, bis(7-methyloctyl) ester

Table S6. GC-MS Chromatogram of Aroeira honey, from Belo Horizonte (Minas Gerais, Brazil).

continuous

Peak#	R.Time	MI	Fragments (%)	Mark	Name
29	44.24	396	97 (100); 57 (99.3); 83(91.4)		1-Heptacosanol
30	51.66	414	43 (100); 107 (98.9); 145 (57.8)	V	Sitosterol
31	44.35	396	97 (100); 57 (99.3); 83(91.4)		1-Heptacosanol
32	44.57	618	57 (100); 71 (89.2); 85 (60.2)	V	Tetratetracontane
33	44.97	562	57 (100); 71 90.2); 85 (70.0)		Tetracontane
34	45.97	562	57 (100); 71 90.2); 85 (70.0)		Tetracontane
35	47.18	410	97 (100); 57 (99.3); 83(91		Octacosanol
36	47.30	396	97 (100); 57 (99.3); 83(91.4)	V	1-Heptacosanol
37	47.45	294	294 (100); 265 (85.0); 237 (50.5)	V	Silane, diethyl (2-ethylphenoxy)
38	47.63	562	57 (100); 71 90.2); 85 (70.0)	V	Tetracontane
39	50.87	324	82 (100); 96 (97.2); 85 (81.0)		13-Docosen-1-ol
40	51.43	382	82 (100); 96 (97.2); 85 (81.0)		1-Hexacosanol
41	51.62	286	97 (100); 57 (99.3); 83(89.4)	V	1-chloro-9-octadecene
42	57.86	380	97 (100); 57 (99.9); 83(90.8)	MI	Nonadecyl trifluoroacetate
43	41.770	618	57 (100); 71 (89.2); 85 (60.2)		Tetratetracontane
